# Association between recurrence risk perception and health behavior in patients with recurrent ischemic stroke in China: the mediation effect of self-efficacy

**DOI:** 10.3389/fpsyg.2025.1608552

**Published:** 2025-10-13

**Authors:** Huan Zhang, Yao Wu, Jiangfeng Pu, Lifang Yuan, Xingyin Tian, Yingying Li

**Affiliations:** ^1^School of Nursing, Guangdong Pharmaceutical University, Guangzhou, China; ^2^Department of Neurology, The Affiliated Yongchuan Hospital of ChongQing Medical University, Chongqing, China; ^3^Department of Neurology, Guangzhou First People's Hospital, Guangzhou, China

**Keywords:** recurrence ischemic stroke, self-efficacy, recurrence risk perception, health behavior, mediation effect, structural equation modeling

## Abstract

**Background:**

This study aims to explore the relationship between recurrence risk perception and health behavior in patients with recurrent ischemic stroke, and the mediating effect of self-efficacy between recurrence risk perception and health behavior.

**Method:**

This cross-sectional study was conducted from May 2023 to November 2023 in China on 280 recurrent ischemic stroke patients. Data on sociodemographic and clinical characteristics, the Recurrence Risk Perception Scale for Stroke Patients (RRPS-SP), the General Self-Efficacy Scale (GSES), and the Health behavior Scale of Stroke Patient (HBS-SP) were included in this study.

**Result:**

There were 266 valid questionnaires (95% effective recovery rate). In correlation analysis, high recurrence risk perception and high self-efficacy were significantly associated with better levels of health behavior (*P* < 0.05). In the multiple linear regression model, after controlling for sociodemographic variables and clinical characteristics information in the *t/F* test (*P* < 0.05), it was found that recurrence risk perception and self-efficacy were significant predictors of health behavior, and that exercise status, Course of Disease, and family history of stroke in the sociodemographic and clinical characteristics information in the present study had a significant effect on health behavior (*P* < 0.05). Self-efficacy partially mediated the relationship between recurrence risk perception and health behavior (95% CI 0.102 to 0.334), with the mediating effect accounting for 37.3% (0.202/0.541)of the total effect.

**Conclusion:**

Recurrence risk perception and self-efficacy were influential factors in promoting health behavior. In addition, the effect of recurrence risk perception on health behavior was mediated by self-efficacy.

## 1 Introduction

The global stroke profile published by the World Stroke Organization (WSO) in 2022 shows that stroke is the second leading cause of death and the third leading combined cause of death and disability in the world (Feigin et al., 2022). And in our country, stroke is the number one cause of death and disability among adults in our country (Wang, 2023). Strokes are divided into haemorrhagic and ischemic strokes, with ischemic strokes being the most common, accounting for about 70% of all strokes (Wang Y. et al., 2019). Ischemic stroke is characterized by a high recurrence rate, and the China Stroke Prevention and Control Report 2021 points out that the recurrence rate of ischemic stroke patients within 5 years after the onset of the disease is 41%, which creates a heavy disease burden and economic burden for patients and their families (Wang, 2023). In addition, a review also reported that the 1-year rehospitalisation rate for ischemic stroke patients after the onset of the disease was 42.5%, with recurrent accidents being the third leading cause of the disease (Zhong et al., 2016). More seriously, functional status after stroke recurrence is often worse than in first stroke (Modrego et al., 2000). But fortunately, 80% of stroke recurrences can be prevented by behavioral changes (Hackam and Spence, 2007).

Good health behaviors can significantly reduce the risk of stroke recurrence. Health behaviors are a set of health-promoting behaviors that an individual undertakes in order to achieve a better state of health (Havigerová et al., 2019). Although it is well established that good health behaviors improve the prognosis of stroke, health behaviors in stroke patients are currently not managed effectively (Xu et al., 2022). It has been noted that about 12.5% of patients are still at risk of stroke recurrence within 12 months due to poor health behavioral status of the patients and there is a tendency for patients to have an increased risk of death after recurrence (Xu et al., 2022). Another cross-sectional study found that 78.4% of stroke patients had moderately low levels of health behavior (Wang X. et al., 2024). It is thus essential to identify the factors influencing the health behaviors of patients with recurrent ischemic stroke. Previous studies have shown that a variety of factors such as exercise status, Course of Disease, family history of stroke, self-efficacy and recurrence risk perception have a significant effect on health behavior in stroke patients (Brouwer-Goossensen et al., 2021; Claeys et al., 2020; Wang X. et al., 2024; Yang et al., 2024; Zhang et al., 2023).

Health behavior related theory suggests that recurrence risk perception is a key factor in promoting behavior change (Li et al., 2022; Weinstein, 1993). recurrence risk perception is the perception of warning features, risk factors, likelihood and severity of recurrence (Lin B. L. et al., 2021). Understanding patients' recurrence risk perception of stroke is important and necessary to translate the recurrence risk perception of stroke and individual risk factors into appropriate behavioral changes (Lin B. et al., 2021). One study confirms that high recurrence risk perception is significantly associated with better health behaviors (Wang X. et al., 2024). However, it has also been found that a low recurrence risk perception is not conducive to healthy behaviors (Ren et al., 2023). In addition, risk perception is not only directly related to an individual's health behavior, but also indirectly affects it through a number of mediating variables (Wang X. et al., 2024). Therefore, more studies are needed to confirm this in order to understand how the recurrence risk perception in ischemic stroke patients affects people's health behavior change.

Self-efficacy is an important determinant of health behavior promotion or impairment. Self-efficacy refers to an individual's confidence and belief in his or her ability to successfully perform a particular task or cope with a particular situation, which influences the choice and persistence of an individual's health behaviors (Bandura, 1977). Study confirms that patients with high self-efficacy experience better health management behaviors (Shuqi et al., 2023). However, in a qualitative study, it was noted that low self-efficacy makes it difficult for patients to make actual behavioral changes, despite adequate knowledge of disease risk (Brouwer-Goossensen et al., 2021). Also ignoring the risk of recurrence may affect stroke survivors' attitudes and confidence in their health prognosis, which in turn may affect patients' health behaviors (Lin B. et al., 2021). However, survivors' neglect of the recurrence risk of stroke is widespread, and the relationship between these beliefs and health behavior change is complex. It has been suggested that self-efficacy has been shown to be an important mediating variable in behavioral change in stroke patients (Nott et al., 2021). Therefore, further validation is needed in order to understand how self-efficacy influences the relationship between recurrence risk perception and health behaviors.

The Health Belief Model (HBM), which links risk perception to health behaviors, was first proposed by social psychologist Hochbaum et al. in 1952, later revised by Becker and other researchers, and is formalized in the HBM framework (Janz and Becker, 1984). It builds on need and motivation theory, cognitive theory, and value-expectancy theory by focusing on people's attitudes and beliefs about health and on the internal and external factors that influence beliefs. The model consists of three components: the individual's health beliefs, cues or intentions for behavior, and constraints on behavior. Among these, the individual's health beliefs include five dimensions: (1) perceived disease susceptibility, (2) perceived disease severity (3) perceived benefits (4) perceived barriers (5) self-efficacy. For stroke patients, individuals who perceive themselves to be at risk of disease and accurately estimate their risk (recurrence risk perception), and who believe (self-efficacy) that behavioral change is effective in reducing the risk of disease, are more likely to take preventive action (health behaviors) (Li et al., 2022). Therefore, it is reasonable to choose the HBM model as the theoretical framework for our study.

**Figure 1:**
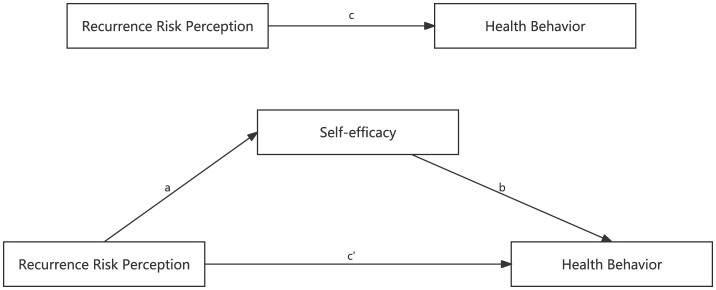
The theoretical framework and structural equation modeling. c: the relationship of Recurrence Risk Perception and Health Behavior; a: the relationship of Recurrence Risk Perception and Self-efficacy; b: the relationship of Self-efficacy and Health Behavior; c′: the relationship of Recurrence Risk Perception and Health Behavior after adopting Self-efficacy as a mediator.

The mediating effect of self-efficacy in the relationship between recurrence risk perception and health behavior in patients with recurrent ischemic stroke has not been demonstrated. The present study aimed to clarify the direct and indirect relationships between these variables and possible causal pathway relationships through health belief modeling (HBM). The theoretical framework of this study and the structural equation modeling (SEM) of the relationship between recurrence risk perception, self-efficacy and health behavior are shown in Figure 1. The following hypotheses were proposed based on the theoretical framework: Hypothesis 1 (H1): recurrence risk perception is positively related to health behavior. Hypothesis 2 (H2): recurrence risk perception is positively related to self-efficacy. Hypothesis 3 (H3): self-efficacy is positively related to health behavior. Hypothesis 4 (H4): self-efficacy partially mediates the relationship between recurrence risk perception and health behavior.

## 2 Materials and methods

### 2.1 Aims

The aim of this study was to investigate the relationship between Recurrence risk perception and self-efficacy and health behavior in patients with recurrent ischemic stroke, and the mediating role of self-efficacy between Recurrence risk perception and health behaviors.

### 2.2 Participants

This study is a cross-sectional investigation employing convenience sampling. Through the medical records system of the Department of Neurology at Guangzhou First People's Hospital in Guangdong Province, China, a list of inpatients meeting diagnostic criteria was retrieved for the period from May 2023 to November 2023. The investigator visited the wards daily, consecutively enrolling all eligible patients according to inclusion and exclusion criteria until the target sample size was achieved. The inclusion criteria were as follows:(a) Patients diagnosed with ischaemic stroke by a neurologist based on clinical symptoms, signs, and results of cranial CT or MRI examinations (Zen, 2019); (b) patients with stable vital signs (Within 1 h prior to data collection, vital signs were measured and recorded by the responsible nurse, falling within the following ranges: heart rate 50–100 beats per min, systolic blood pressure 90–160 mmHg, diastolic blood pressure 60–100 mmHg, respiratory rate 16–20 breaths per min, and oxygen saturation ≥ 95%), normal communication, and ability to cooperate with the investigation (c) Age ≥ 18 years old; (d) number of episodes ≥ 2; (e) Course of Disease ≥1 month; and (f) patients' informed consent. Exclusion criteria: (a) comorbidity with severe cardiac, hepatic, renal dysfunction or other malignancies; (b) history of severe mental illness or family history of severe mental illness. According to Kendall's method for determining sample size in multivariate analysis, the sample size should be 5 to 10 times the number of study factors (Ni et al., 2010). This study includes 29 influencing factors: three dimensions of RRPS-SP, five dimensions of GSES, six dimensions of HBS-SP, and 15 sociodemographic and clinical characteristics items. The calculated sample size range was 145–290 cases. To account for potential non-response, missing data, and loss to follow-up, an additional 20% sample expansion was applied, resulting in a final survey sample size range of 174–348 cases. Structural equation modeling requires a minimum sample size of 200 cases. This study distributed questionnaires to 280 participants. After excluding non-respondents and invalid samples, the final collected sample comprised 266 cases, yielding a questionnaire return rate of 95%.

### 2.3 Data collection

The collection of questionnaires was conducted with the consent of hospital and departmental administrators, with the researcher personally distributing paper questionnaires to patients on a one-to-one basis. Prior to distribution, the purpose of the study and relevant precautions were thoroughly explained to participants. Following the signing of informed consent forms, questionnaires were provided for self-completion. For aphasic patients unable to complete written questionnaires, researchers trained in a standardized procedure conducted structured interviews. Each question was read aloud, with response options presented via cards. Participants indicated their choice through pointing, eye contact, or gestures, and researchers recorded responses accordingly. Upon completion, researchers immediately checked for missing data. For omitted, redundant, or questionable responses, they promptly explained the issue to the patient, requesting completion or correction as required. After a final verification, the questionnaire was collected.

### 2.4 Measurements

The sociodemographic and clinical characteristics questionnaire was developed by the researcher based on the literature review and included variables such as gender, BMI index, age, occupation, education level, spouse status, place of residence, monthly income, living alone or not, cigarette smoking, drinking alcohol, exercise activity, number of episodes, course of disease, and family history of stroke.

RRPS-SP is a tool used to assess stroke patients' perception of their risk of disease recurrence. It was developed by Lin B. L. et al. (2021). The scale consists of two parts, the first part is the perception of the likelihood of recurrence and consists of 3 items. Since it could not be scored and reliability and validity tests were not conducted, only the second part of the scale was used in this study, which consisted of 3 dimensions and 17 items, respectively: the dimension of perceived severity consisted of 7 items (referring to the patient's perception of the harm of recurrence on daily activities, mood, cognition, degree of impairment, and frequency of recurrence); The perceived behavioral risk factors dimension consisted of 6 items (referring to patients' perceptions of behavioral risk factors for preventing stroke recurrence, such as intake of vegetables, fruits, and salt, as well as the effect of risk factors such as smoking, alcohol consumption, and exercise on recurrence); and dimension of perceived illness risk factors consisted of 4 items (including patients' perceptions of disease risk factors such as hyperlipidaemia, hyperglycaemia, hypertension and atrial fibrillation on stroke recurrence). A Likert 3 scale was used, scoring 1 to 3 from “disagree” to “agree,” with a total score ranging from 17 to 51. A higher total score represents a higher level of recurrence risk perception for the patient. This study has been authorized by the original authors of the scale.

GSES is a psychometric tool that assesses an individual's level of self-confidence and sense of competence in dealing with various challenges in daily life. The scale was developed by Schwarzer's team in Germany and translated and revised into Chinese by Zhang's team (Cheung and Sun, 1999). It consists of 5 dimensions and 10 items, including Perceived Effort, Innate Ability, Environmental Perception, Goal Attainment, and Self-forecasting. Each dimension has 2 items. Each entry describes a situation that may be encountered in daily life, and subjects are asked to rate the description of each entry according to their own reality. The GSES uses a Likert 4 scale, scoring 1 to 4 from “not at all correct” to “completely correct”, with a total score ranging from 10 to 40. The higher the total score, the higher the sense of self-efficacy. This study has been authorized by the original authors of the scale.

HBS-SP is a scale to assess the level of health behavior of stroke patients. The scale was developed by Prof Wan Li-Hong's team (Wan et al., 2017). The scale consists of 6 dimensions and 25 items. The Exercise Dimension consists of 6 items dealing with exercise duration, frequency, type, intensity, programme and motivation. The Responsibility Dimension consists of 3 items, including monitoring heart rate while paying attention to exercise and paying attention to the ingredient list on food package labels. The Instructions Dimension consists of 4 items, which mainly include the appropriate intake of salt, sugar and oil as prescribed by the doctor and the monitoring of blood pressure. The Nutrition dimension consists of 6 items, i.e., moderate consumption of cereals, fruits, meat, eggs, milk, soya products, etc. The Nutrition dimension consists of 2 items, i.e., moderate consumption of tobacco and alcohol. The dimension of smoking and drinking has 2 items, i.e., moderate smoking and drinking. The medication taking dimension had 4 items, namely knowledge of medication and adherence to medication. Each item was scored using the Likert4 scale, including “never”, “sometimes”, “often”, and “always”, with scores ranging from 1 to 4, respectively. The scores were reversed for smoking, drinking and medication taking. Higher scores indicate higher levels of health behavior. An average score of 2.5 (between “sometimes” and “often”) is considered a moderate level of health behavior. This study has been authorized by the original authors of the scale.

### 2.5 Data analysis

Data were analyzed using IBM SPSS Statistics 27.0 and AMOS Graphics 26.0 software. Information on participants' sociodemographic variables and clinical characteristics was examined through descriptive statistics of frequencies, percentages, means, standard deviations, *t/F* tests, and score ranges. Pearson's correlation was used to test the relationship between recurrence risk perception, self-efficacy and health behavior. Multiple linear regression was used to assess whether recurrence risk perception and self-efficacy significantly influence health behavior. Socio-demographic variables and clinical information (education level, place of residence, monthly income, smoking, exercise status, course of disease, family history of stroke) were examined as significant predictors of health behaviors of stroke patients in this study according to *t/F* test. Multiple linear regression analysis was established in three steps with health behavior as the dependent variable. In the first step, sociodemographic variables (education level, place of residence, monthly income, smoking, exercise status, course of disease, family history of stroke) were included in Model I as controlled variables. The second step was to add the recurrence risk perception as a dependent variable to Model II based on Model I. In the third step, self-efficacy was added to model II to construct model III. Finally, SEM and Bootstrap methods (5,000 repetitions) were used to test the mediating role of self-efficacy on recurrence risk perception and health behavior. A two-tailed significance level of 0.05 was used for statistical analysis.

### 2.6 Validity and reliability

The Recurrence Risk perception Scale has been applied to Chinese stroke patients, with a Cronbach's α of 0.850 for the second part (Wang X. et al., 2024). In our study, the Cronbach's α value for the second part of the scale was 0.775, and the results of the validation analyses indicated that the scale was structurally stable. The GSES showed good structural validity and internal consistency reliability across gender, occupation, and disease in China. The Cronbach's α value for the GSES was reported to be 0.804 (Wang S. et al., 2024). In the present study, the Cronbach's α value for the GSES was 0.885, indicating good reliability. In addition, a previous report reported a value of 0.878 for Cronbach's α for the HBS-SP (Wang X. et al., 2024). In the present study, the Cronbach's α value for the HBS-SP was 0.937, indicating that the scale has good reliability.

## 3 Results

### 3.1 Descriptive statistics of the measurements

A total of 280 questionnaires were obtained for this study. After excluding invalid questionnaires with a completion rate of less than 90 percent, 266 valid questionnaires returned finally. The age of the stroke patients ranged from 26 to 88 years with a mean age of 65.40 years (standard deviation 10.25 years). Details of the participants are detailed in Table 1.

**Table 1:** Univariate analysis of demographic and clinical characteristics and health behaviors in recurrent ischemic stroke patients (*N* = 266).

**Factors**	**Group**	**N(%)**	**HBS-SP (Mean ±SD)**	***t/F* test**	***P* value**
Gender	Male	190(71.4)	55.52 ± 12.99	0.979^a^	0.328
	Female	76(28.6)	57.32 ± 14.73		
BMI index	<18.5	6(2.3)	54.83 ± 12.77	0.524^b^	0.666
	18.5~23.9	125(47.0)	57.13 ± 13.89		
	24.0~27.9	103(38.7)	54.96 ± 12.10		
	≥28.0	32(12.0)	55.44 ± 16.43		
Age	18–59	65(24.4)	55.71 ± 13.87	0.740 ^b^	0.478
	60–74	151(56.8)	55.48 ± 12.91		
	≥75	50(18.8)	58.12 ± 14.81		
Occupation	Enterprise	12(4.5)	60.54 ± 4.78	1.638 ^b^	0.165
	Public institution	14(5.3)	53.43 ± 14.67		
	Freelance	23(8.6)	53.96 ± 15.05		
	Farmer	21(7.9)	50.57 ± 10.74		
	Unemployed/retired	196(73.7)	56.77 ± 13.21		
Educational level	Primary school or below	81(30.5)	53.49 ± 12.50	3.960 ^b^	0.004
	Middle school	82(30.8)	55.18 ± 13.34		
	Senior high school	79(29.7)	56.71 ± 13.29		
	Undergraduate college	13(4.9)	67.08 ± 13.99		
	Undergraduate and above	11(4.1)	63.18 ± 15.87		
Spouse status	No	25(9.4)	52.52 ± 12.07	1.369 ^a^	0.172
	Have	241(90.6)	56.4 ± 13.62		
Place of residence	Urban towns	48(18.0)	51.88 ± 11.95	2.377 ^a^	0.018
	City	218(82.0)	56.95 ± 13.68		
Monthly income(RMB)	≤2000	14(5.3)	52.21 ± 11.39	5.427 ^b^	0.001
	2001–4000	85(32.0)	55.47 ± 12.30		
	4001–6000	110(41.4)	53.84 ± 12.94		
	>6000	57(21.4)	62.05 ± 15.16		
Living alone or not	No	35(13.2)	53.26 ± 13.48	1.307 ^a^	0.192
	Yes	231(86.8)	56.45 ± 13.49		
Cigarette smoking	No	179(67.3)	57.35 ± 14.15	2.458 ^a^	0.022
	Yes	87(32.7)	53.32 ± 11.69		
Drinking alcohol	No	222(83.5)	56.23 ± 13.93	0.540 ^b^	0.583
	Sometimes	31(11.7)	56.19 ± 10.64		
	Frequently	13(4.9)	52.23 ± 12.34		
Exercise status	Frequently	136(51.1)	59.22 ± 13.24	8.185 ^b^	<0.001
	Insufficient	113(42.5)	52.62 ± 13.18		
	Never	17(6.4)	53.24 ± 12.17		
Number of episodes	Twice	197(74.1)	56.31 ± 13.46	2.089 ^b^	0.126
	Three times	61(22.9)	54.08 ± 12.90		
	Four times or more	8(3.0)	64.00 ± 17.18		
Course of disease	1 to 3 month	20(7.5)	50.35 ± 13.12	4.770 ^b^	<0.001
	3 to 6 month	21(7.9)	48.76 ± 10.78		
	6 month to 1 year	31(11.7)	53.10 ± 12.94		
	1 to 5 years	108(40.6)	56.29 ± 12.90		
	More than 5 years	86(32.3)	59.87 ± 13.98		
Family history of stroke	No	225(84.6)	54.53 ± 13.13	4.403 ^a^	<0.001
	Yes	41(15.4)	64.29 ± 12.66		

^a^t test; ^b^F test; BMI, Body Mass Index; RMB, Renminbi.

### 3.2 Descriptive statistics of RRPS-SP, GSES, and HBS-SP

The mean, standard deviation and range of scores for recurrence risk perception, self-efficacy and health behavior are shown in Table 2. The mean score and standard deviation for recurrence risk perception was (40.10 ± 5.08). The mean score and standard deviation of self-efficacy was (27.09 ± 6.95). The mean score and standard deviation of health behavior was (56.03 ± 13.50). Among our recruited stroke patients, both recurrence risk perception and self-efficacy were at moderately high levels, as the mean scores for item entries on RRPS-SP and ESES were 2.36 and 2.71. Health behavior were moderately low, as the mean score for item entries on the HBS-SP scale was 2.24.

**Table 2:** Descriptive statistics of RRPS-SP, GSES, and HBS-SP (N = 266).

**Variables**	**Items**	**Range of score**	**Mean**	**SD**
Perceived severity	7	7–21	17.32	2.62
Perceived behavioral risk factors	6	6–18	14.07	2.46
Perceived illness risk factors	4	4–12	8.70	1.78
RRPS-SP	17	17–51	40.10	5.08
Perceived effort	2	2–8	5.63	2.01
Innate ability	2	2–8	5.29	1.73
Environmental perception	2	2–8	5.42	1.77
Goal attainment	2	2–8	5.59	2.00
Self-forecasting	2	2–8	5.16	1.82
GSES	10	10–40	27.09	6.95
Exercise	6	6–24	13.58	4.13
Responsibility	3	3–12	4.46	1.70
Instructions	4	4–16	8.99	3.01
Nutrition	6	6–36	14.65	4.24
Smoking and drinking	2	2–8	4.67	1.92
Medication taking	4	4–16	9.69	3.36
HBS-SP	25	25–100	56.03	13.50

RRP-SP, Recurrence Risk Perception Scale for stroke patients; GSES, General Self-Efficacy Scale; HBS-SP, Health behavior Scale for stroke patients.

### 3.3 Correlation analysis of recurrence risk perception, self-efficacy, and health behavior

Pearson's correlation analysis was used to study the correlation between recurrence risk perception, self-efficacy and health behavior in stroke patients, as shown in Table 3. The results showed that recurrence risk perception was positively correlated with health behavior (*r* = 0.365, *p* < 0.01), Self-efficacy is positively correlated with health behavior (*r* = 0.429, *p* < 0.01), recurrence risk perception is positively associated with Self-efficacy (*r* = 0.377, *p* < 0.01), which implies that there is a positive correlation between recurrence risk perception, self-efficacy and health behavior in stroke patients.

**Table 3:** Correlation between Recurrence risk perception, Self-efficacy, and Health behavior (N = 266).

	**1**	**2**	**3**	**4**	**5**	**6**	**7**	**8**	**9**	**10**	**11**	**12**	**13**	**14**	**15**	**16**	**17**
1 Perceived severity	-																
2 Perceived behavioral risk factors	0.311^a^	-															
3 Perceived illness risk factors	0.237^a^	0.395^a^	-														
4 RRPS-SP	0.750^a^	0.783^a^	0.665^a^	-													
5 Perceived effort	0.154^b^	0.388^a^	0.302^a^	0.373^a^	-												
6 Innate ability	0.103	0.291^a^	0.177^a^	0.256^a^	0.418^a^	-											
7 Environmental perception	0.104	0.324^a^	0.201^a^	0.281^a^	0.434^a^	0.432^a^	-										
8 Goal attainment	0.098	0.310^a^	0.200^a^	0.271^a^	0.437^a^	0.495^a^	0.476^a^	-									
9 Self-forecasting	0.097	0.240^a^	0.126^b^	0.211^a^	0.410^a^	0.429^a^	0.433^a^	0.453^a^	-								
10 GSES	0.150^b^	0.419^a^	0.273^a^	0.377^a^	0.737^a^	0.735^a^	0.739^a^	0.778^a^	0.729^a^	-							
11Exercise	0.116	0.303^a^	0.195^a^	0.275^a^	0.300^a^	0.249^a^	0.268^a^	0.300^a^	0.174^a^	0.349^a^	-						
12Responsibility	0.106	0.266^a^	0.140^b^	0.233^a^	0.254^a^	0.234^a^	0.300^a^	0.371^a^	0.206^a^	0.369^a^	0.395^a^	-					
13Instructions	0.255^a^	0.304^a^	0.280^a^	0.377^a^	0.278^a^	0.249^a^	0.231^a^	0.207^a^	0.151^b^	0.300^a^	0.443^a^	0.434^a^	-				
14Nutrition	0.078	0.320^a^	0.212^a^	0.270^a^	0.246^a^	0.306^a^	0.284^a^	0.268^a^	0.193^a^	0.347^a^	0.436^a^	0.400^a^	0.448^a^	-			
15Smoking and drinking	0.163^a^	0.271^a^	0.184^a^	0.280^a^	0.260^a^	0.216^a^	0.279^a^	0.302^a^	0.113	0.317^a^	0.429^a^	0.448^a^	0.442^a^	0.457^a^	-		
16Medication taking	0.058	0.201^a^	0.128^b^	0.172^a^	0.162^a^	0.248^a^	0.193^a^	0.168^a^	0.051	0.220^a^	0.442^a^	0.429^a^	0.444^a^	0.476^a^	0.386^a^	-	
17HBS-SP	0.168^a^	0.383^a^	0.264^a^	0.365^a^	0.340^a^	0.349^a^	0.348^a^	0.353^a^	0.202^a^	0.429^a^	0.762^a^	0.639^a^	0.727^a^	0.781^a^	0.668^a^	0.741^a^	-

RRP-SP, Recurrence Risk Perception Scale for stroke patients; GSES, General Self-Efficacy Scale; HBS-SP, Health behavior Scale for stroke patients.

^*a*^*P* < 0.01;^*b*^*P* < 0.05.

### 3.4 The mediation effect of self-efficacy on the relationship of recurrence risk perception and health behavior

A multiple linear regression model was developed using regression with socio-demographic variables and clinical characteristic information from the *t/F* test (*P* < 0.05) as control variables; health behaviors as dependent variables; and recurrence risk perception and self-efficacy as predictor variables (See Table 4). The results of Model I (*F* = 8.331, *P* < 0.001, R2 = 0.162) indicated that sociodemographic variables and clinical characteristic information explained 16.2% of the variance in health behavior. Among these, exercise status, Course of Disease, and family history of stroke were significant predictors of health behavior. In model II (*F* = 10.208, *p* < 0.001, R2 = 0.218), controlling for socio-demographic variables and clinical characteristics information, recurrence risk perception was added as an independent variable and all of them explained 21.8% of the variance in health behavior, of which 5.6% was explained by recurrence risk perception. In model II, recurrence risk perception was a significant predictor of health behavior, and exercise status and family history of stroke had a significant effect on health behavior. In Model III (*F* = 13.729, *P* < 0.001, R2 = 0.302), self-efficacy was included in the regression model and explained an additional 8.4% of the variance in health behavior. In model III, recurrence risk perception and self-efficacy were significant predictors of health behavior, with exercise status, Course of Disease, and family history of stroke having a significant effect on health behaviors in this study.

**Table 4:** Multiple linear regression analysis of factors influencing health behavior in patients with recurrent ischemic stroke (N = 266).

**Model**	**Variables**	**B(SE)**	**Beta**	***t***	***p***	***F* value**	**AdjustedR^2^**
Model I	Educational level	1.428(0.772)	0.112	1.850	0.065	8.331^a^	0.162
	Residence	1.060(1.231)	0.055	0.861	0.390		
	Monthly income	0.547(1.024)	0.034	0.535	0.593		
	Cigarette smoking	−1.925(1.652)	−0.067	−1.165	0.245		
	Exercise status	−4.079(1.257)	−0.185	−3.245	0.001		
	Course of disease	1.990(0.660)	0.175	3.015	0.003		
	Family history of stroke	7.657(2.164)	0.205	3.538	<0.001		
Model II	Educational level	1.015(0.752)	0.080	1.349	0.178	10.208^a^	0.218
	Residence	1.114(1.190)	0.058	0.936	0.350		
	Monthly income	0.654(0.990)	0.041	0.661	0.509		
	Cigarette smoking	−1.966(1.597)	−0.068	−1.231	0.219		
	Exercise status	−3.382(1.225)	−0.154	−2.761	0.006		
	Course of disease	1.291(0.657)	0.113	1.963	0.051		
	Family history of stroke	6.370(0.657)	0.113	1.963	0.051		
	Recurrence risk perception	0.679(0.155)	0.255	4.385	<0.001		
Model III	Educational level	0.865(0.711)	0.068	1.217	0.225	13.729^a^	0.302
	Residence	0.820(1.125)	0.042	0.729	0.467		
	Monthly income	1.022(0.937)	0.063	1.091	0.276		
	Cigarette smoking	−1.600(1.510)	−0.056	−1.060	0.290		
	Exercise status	−2.758(1.162)	−0.125	−2.373	0.018		
	Course of Disease	1.494(0.622)	0.131	2.402	0.017		
	Family history of stroke	5.569(2.000)	0.149	2.785	0.006		
	Recurrence risk perception	0.374(0.156)	0.141	2.401	0.017		
	Self-efficacy	0.617(0.109)	0.318	5.660	<0.001		

The variable of Recurrence risk perception and Self-efficacy was entered as a continuous variable. The other sociodemographic variables were entered as categorical variables. Educational level (1:Primary school or below, 2:Middle school, 3:Senior high school, 4:Undergraduate college, 5: Undergraduate and above); Residence (1:Urban Towns, 2:City); Monthly income (1: ≤ 2000, 2:2001~4000, 3:4001~6000, 4: > 6000). Cigarette smoking (1:No, 2:Yes). Exercise status (1:Frequently, 2:Insufficient, 3:Never). Course of Disease (1:1 to 3 month, 2:3 to 6 month, 3:6 month to 1 year, 4:1 to 5 years, 5:More than 5 years). Family history of stroke (0:No, 1:Yes).^*a*^*P* < 0.001.

**Figure 2:**
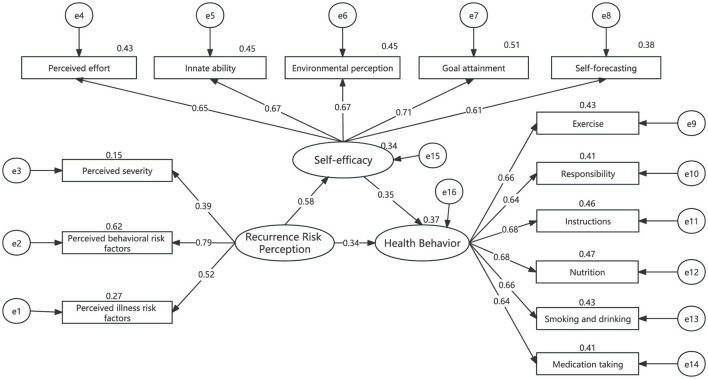
Pathways of the structural equation model (SEM) (*N* = 266). All path coefficients were standardized estimates.

We used SEM to test the mediating effect of self-efficacy. The path analysis of SEM is shown in Figure 2. The results showed that recurrence risk perception directly affected health behavior (β = 0.339, *P* = 0.002) and self-efficacy (β = 0.580, *P* < 0.001); meanwhile, self-efficacy was also directly related to health behavior (β = 0.349, *P* < 0.001), as detailed in Table 5. In addition, the recurrence risk perception was indirectly associated with health behavior through the mediator of self-efficacy (β = 0.580^*^ 0.349 = 0.202, *p* = 0.002), as detailed in Table 6. The results indicated that the fit of SEM was accepted and significant. After establishing the presence of self-efficacy as a mediating variable, further validation of the significance of the mediating effect is needed. The standardized direct, indirect and total effects of recurrence risk perception, self-efficacy and health behavior are shown in Table 6. The Bootstrap 95% confidence interval for the indirect effect between recurrence risk perception and health behavior did not include 0 (0.102 to 0.334), suggesting that self-efficacy has a significant mediating effect in the relationship between recurrence risk perception and health behavior. We also found that the partial mediating effect of self-efficacy on health behavior accounted for 37.3% of the total effect (0.202/0.541). Assessing the fit of the model to the data by calculating the goodness-of-fit index shows that CMIN/degree of freedom (χ2/df) = 1.077; Root mean square error of Approximation (RMSEA) = 0.017; goodness-of fit index (GFI) = 0.959; adjusted goodness-of-fit index (AGFI) = 0.942; Request for Information (RFI) = 0.910; normalized fit index (NFI) = 0.927; incremental fit index (IFI) = 0.994; comparative fit index (CFI) = 0.994;tucker-lewis index (TLI) = 0.993. All pathway coefficients of SEM were significant at the level of 0.05. A SEM was considered acceptable when χ2/df was < 3.000; RMSEA was < 0.080; GFI, AGFI, RFI, NFI, IFI, CFI, and TLI were ≥0.900; indicating that the fit of SEM was accepted and significant.

**Table 5:** The standardized path coefficients of the structural equation model among recurrence risk perception, self-efficacy, and health behavior.

**Path analysis testing**	**β**	**SE**	***t***	***p***
SE←RRP	0.580	0.162	5.099	<0.001
HB←SE	0.349	0.212	3.406	<0.001
HB←RRP	0.339	0.323	3.082	0.002

SE, Self-efficacy; RRP, recurrence risk perception; HB, health behavior.

**Table 6:** The results of bootstrap mediation analysis among recurrence risk perception, self-efficacy, and health behavior.

**Model pathway**	**Point estimate**	**95%CI**	***P***	**Effect size proportion**
Direct effect (RRP → HB)	0.339	0.146–0.555	0.001	62.7%
Indirect effect (RRP → SE → HB)	0.202	0.102–0.334	0.002	37.3%
Total effect (RRP → HB)	0.541	0.391–0.671	0.001	100%

SE, Self-efficacy; RRP, recurrence risk perception; HB, health behavior. Number of bootstrap samples: 5.

## 4 Discussion

We found that 266 patients with recurrent ischemic stroke had a mean score of less than 2.5 on the Health behavior Survey, implying that their health behaviors were in the lower-middle range. This result is consistent with the findings of previous studies (Liu et al., 2021; Wang X. et al., 2024). Firstly, a growing body of research suggests that lifestyle changes are essential for secondary prevention of stroke (Govori et al., 2024). Secondly, it has also been shown that improving recurrence risk perceptions promotes survivors' willingness to adopt healthy behaviors and allows survivors to translate their willingness into long-term behaviors with a high sense of self-efficacy (Yenit et al., 2023). In addition, many theories related to health behavior suggest that people's perception of risk is a key factor influencing their willingness to change their behavior (Wang et al., 2022). Risk perception and self-efficacy may also be predictors of people choosing healthy behaviors and engaging in preventive behaviors (Ferrer et al., 2018; Sheeran et al., 2016). Our study confirms that high recurrence risk perception and high self-efficacy are closely related to and influence the formation of health behavior in patients with recurrent ischemic stroke, which provides a practical reference for the secondary prevention of recurrent ischemic stroke patients. Therefore, this study will explore the correlation between (1) recurrence risk perception, self-efficacy and health behavior, and (2) the mediating role of self-efficacy between recurrence risk perception and health behavior.

### 4.1 Correlation analysis

The results of this study found a positive correlation between recurrence risk perception and health behavior scores in patients with recurrent ischemic stroke, which is consistent with previous findings (Wang X. et al., 2024). Recurrence risk perception is an important factor in promoting healthy behaviors in survivors (Ren et al., 2023). When patients perceive themselves to be at a higher risk of recurrence, they tend to be more willing to adopt healthy lifestyles and behaviors, such as regular exercise, healthy diet, regular monitoring of blood pressure and blood glucose, cessation of smoking and limitation of alcohol consumption, and adherence to medication to reduce the risk of stroke recurrence. In addition, a clear understanding of the risk of recurrence can enable patients to feel more in control of their disease and choose more appropriate health behaviors (Boden-Albala et al., 2011). Therefore, understanding the relationship between recurrence risk perceptions and health behaviors is more helpful for caregivers and health educators to develop more effective intervention strategies to enhance patients' health behaviors and prevent recurrence.

The results of the present study confirmed that recurrence risk perceptions were positively associated with self-efficacy in patients with recurrent ischaemia. This finding is consistent with other studies (Sheeran et al., 2014; Yenit et al., 2023). Willingness to change behavior only occurs when patients are aware of their risk of recurrence and have a response to it (Brouwer-Goossensen et al., 2021). Multiple studies have also noted that survivors who perceive themselves to be at high risk of recurrence may be more active in seeking information and learning strategies to manage their risk, and that this learning process and successes, whether direct or indirect, increase survivors' confidence in their own abilities (Bandura, 1977; Sheeran et al., 2014). Therefore, by increasing survivors' awareness of the risk of disease recurrence and improving their self-efficacy, caregivers and health educators can be more effective in promoting patient health behaviors and reducing the risk of disease recurrence.

The results of this study show that self-efficacy is positively correlated with health behaviors in patients with recurrent ischemic stroke, which is consistent with the findings of further studies (Brouwer-Goossensen et al., 2021; Szczepańska-Gieracha and Mazurek, 2020). Self-efficacy as a coping factor is the most important determinant of health behavior (Brouwer-Goossensen et al., 2021). A growing body of research suggests that patients with higher self-efficacy have stronger beliefs, have higher self-goals and provide extra energy for action, while patients with low self-efficacy are associated with psychological distress, and a lack of self-efficacy can completely cripple a person's ability to take action and lead to a decrease in the patient's adherence to health behavior (Bandura, 1977; Szczepańska-Gieracha and Mazurek, 2020). This could also be explained by the fact that patients with high self-efficacy are more confident in their ability to manage stress without experiencing psychological distress and are more likely to adopt health-promoting behaviors.

### 4.2 Mediation effect

Health behavior in stroke patients are associated with many influencing factors such as exercise status, disease duration, family history of stroke, etc (Claeys et al., 2020; Yang et al., 2024; Zhang et al., 2023). According to the results in Model I, good exercise, longer course of Disease and family history of stroke are important factors influencing health behavior in patients with recurrent ischemic stroke. Possible explanations for this are, firstly, that patients who are able to exercise on a regular basis usually have greater self-discipline, and this self-discipline tends to extend to other health behaviors. Secondly, in the early stages of the disease, patients did not know much about their own disease, but as the course of stroke increased, patients had a more comprehensive understanding of the risk factors for stroke and the content of care, and also recognized the importance of health behaviors in the management of the disease, which led to a more conscientious adoption of positive health behaviors; In addition, the longer the course of the disease, the more the complications associated with the disease appear, the more the patients feel the physical and mental suffering caused by the disease, which makes them more willing to adopt positive health behaviors to change the painful status quo, so the longer the duration of the disease, the higher the level of health behaviors of the patients. Finally, patients with a family history of stroke are usually more aware of their risk of developing the disease, and this awareness motivates them to pay more attention to their health status and to take proactive preventive measures to reduce the likelihood of stroke. In Model II and III, recurrence risk perception and self-efficacy had a significant effect on health behavior, with recurrence risk perception explaining 5.6% of the variance and self-efficacy explaining 8.4% of the variance. In addition, the SEM results confirmed the partial mediating role of self-efficacy between recurrence risk perceptions and health behaviors. Therefore, in addition to self-efficacy, there are many variables moderating the relationship between recurrence risk perceptions and health behaviors, such as behavioral decision-making, emotional reactions, etc., which need to be further explained and validated (Lin et al., 2022, 2024).

Our findings suggest that recurrence risk perceptions not only directly affect the health behaviors of patients with recurrent ischemic stroke, but also indirectly affect the level of health behaviors through patients' self-efficacy in the face of illness. This finding is consistent with previous studies on the relationship between disease perceptions, psychological perceptions, self-efficacy and health behaviors. For example, it has been suggested that self-efficacy mediates the perceived severity of non-communicable diseases (NCDs) and the relationship between perceived impairment and health behaviors (Tshuma et al., 2017). Several studies have also found that self-efficacy and body condition awareness are positive predictors of physical activity in older adults, with self-efficacy mediating the relationship between body condition awareness and physical activity (Van Stralen et al., 2011). In addition, other studies have confirmed that breast cancer cognitive impairment can have an indirect effect on health behaviors through the mediating role of self-efficacy (Kim et al., 2024). Stroke has a high recurrence rate, and the severity of symptoms and increased risk of death after recurrence can lead to severe psychological distress in patients about their disease state. The recurrence risk perception of disease can motivate patients to take preventive measures, such as making lifestyle changes or seeking medical help. However, only 5.6% of the variance in health behavior in this study was explained by recurrence risk perception and 14.0% by a combination of recurrence risk perception and self-efficacy, so recurrence risk perception alone is not sufficient to directly lead to changes in health behavior. Whether or not an individual will take action depends largely on whether or not they perceive themselves to be capable of completing those actions, and this is where self-efficacy plays a key role. Therefore, there is a need to design effective interventions based on self-efficacy. By improving patients' self-efficacy, caregivers and health educators can be more effective in helping patients translate perceived recurrence risk into positive health behaviors, thereby improving health outcomes.

In conclusion, our findings provide practical support for HBM and validate the existence and acceptability of the research hypotheses. Self-efficacy, as a mediating variable, increases the likelihood that individuals will translate recurrence risk perception into actual health behavior. The HBM framework in this study has similarities with the Social Cognitive Theory and the Theory of Planned behavior. The commonality lies in the fact that these theories aim to promote health behavior change in people with chronic diseases. The differences lie in the entry points, measures and methods chosen to achieve this goal. HBM focuses on individual perceptions of disease susceptibility and severity, as well as assessment of behavioral benefits and barriers, emphasizing the role of self-efficacy in promoting health behaviors (Janz and Becker, 1984). Social cognitive theory emphasizes observational learning, role modeling effects and self-regulation. While self-efficacy is a core concept, there is a greater emphasis on enhancing self-efficacy by observing the behavior and outcomes of others (Bandura, 2001). The Theory of Planned behavior emphasizes that an individual's behavioral intentions are the primary determinants of behavior and are influenced by attitudes, subjective norms and perceived behavioral control, similar to self-efficacy, but with a greater focus on external environmental factors (Ajzen, 2011). Therefore, from the results of this study, the structure of the HBM is clearer and more consistent with the research hypothesis of this study. At the same time, the model can understand and intervene in individual differences, such as differences in perceived susceptibility and severity among different individuals. The Health Belief Model provides an effective theoretical framework for understanding and intervening in health behaviors.

### 4.3 Implications for practice

Based on the results of this study, the research intervention we designed is actually supported by HBM. The practical implications are to identify entry points for health behavior interventions by adopting an approach to improve recurrence risk perception and self-efficacy in patients with recurrent ischemic stroke. For example, (1) Enhancement of recurrence risk perception, including personalized risk assessment, health education, and regular follow-up assessment. (2) Enhancing self-efficacy, including skills training, social support and role models, and psychological support. Personalized risk assessment, health education, regular follow-up and assessment, skills training, social support, psychological support, etc., are all key strategies. They not only help patients better understand and manage their risks, but also increase their confidence in adopting and maintaining healthy behaviors, thereby improving overall health outcomes. This is an important guide for the field of nursing practice and health promotion.

### 4.4 Limitations

It should be noted that this study has several limitations: (1) Due to practical constraints, the imaging diagnostic criteria for patients included both CT and MRI scans. While this approach better reflects clinical practice, it did not uniformly employ MRI—which offers higher sensitivity—as the diagnostic standard. Future research may focus more closely on a homogeneous stroke cohort confirmed by MRI. Furthermore, while efforts were made to include aphasic patients and alternative communication methods were employed for data collection, the quality of data from this cohort may still differ from that of patients with intact speech function, representing a potential limitation of this study. (2) Although our study confirms a significant correlation between recurrence ischemic stroke patients' recurrence risk perception and their health behavior, this cross-sectional investigation cannot establish causality between the two. Furthermore, patients' recurrence risk perception exhibits dynamic characteristics, fluctuating markedly with the number of stroke episodes. Therefore, a longitudinal design is required to explore the dynamic characteristics of recurrence risk perception. (3) This study was conducted exclusively within the neurology ward of a tertiary hospital in Guangzhou, Guangdong Province, and its findings may not be universally applicable to all patients with recurrence ischemic stroke. Furthermore, the variables of recurrence risk perception and self-efficacy explained less than 15% of the variance in health behavior, and self-efficacy demonstrated only partial mediating effects. Consequently, further research is required to explore other potential mediating factors linking recurrence risk perception with health behavior in patients with recurrence ischemic stroke. (4) The relatively advanced age of recurrence ischemic stroke patients included in this study may limit the generalisability of the findings. Future research should design multicentre longitudinal studies with a particular focus on younger patient cohorts.

## 5 Conclusion

In conclusion, this study demonstrated that recurrence risk perception and self-efficacy were significantly associated with health behaviors in patients with recurrent ischemic stroke, and that self-efficacy partially mediated recurrence risk perception and health behaviors in patients with recurrent ischemic stroke. The findings also suggest that patients with high self-efficacy are more likely to adopt preventive health behaviors when patients with recurrent ischemic stroke have a positive perception of their risk of disease recurrence. Targeted interventions focusing on improving individuals' self-efficacy, particularly in the context of a high recurrence risk perception, are therefore needed to promote positive health behavior change and thereby improve overall health, which is an important guide for nursing practice and public health policy development. Given the importance of recurrence risk perceptions and self-efficacy, it is recommended that these factors be included in the assessment process of patients with recurrent ischemic stroke, both during hospitalization and out-of-hospital follow-up. In addition, further research should explore the impact of dynamic trends in recurrence risk perceptions on health behaviors and explore other mediators between recurrence risk perceptions and health behaviors.

## Data Availability

The original contributions presented in the study are included in the article/supplementary material, further inquiries can be directed to the corresponding author/s.
